# Efficacy of transforaminal lumbar interbody fusion in the treatment of double-level lumbar spondylolisthesis with sagittal imbalance

**DOI:** 10.1186/s12891-022-06018-w

**Published:** 2022-12-01

**Authors:** Haopeng Luan, Yao Wang, Kai Liu, Weibin Sheng, Qiang Deng

**Affiliations:** 1 Department of Spine Surgery, The First Affiliated Hospital of Xinjiang Medical University, Urumqi, 830054 Xinjiang China; 2 Department of Trauma and Microreconstructive Surgery, The First Affiliated Hospital of Xinjiang Medical University, Urumqi, 830054 Xinjiang China

**Keywords:** Spondylolisthesis, Transforaminal lumbar interbody fusion, Sagittal parameter, Double-level

## Abstract

**Objective:**

To analyze the clinical efficacy of transforaminal lumbar interbody fusion (TLIF) in the treatment of continuous double-level lumbar spondylolisthesis with sagittal imbalance.

**Methods:**

The clinical data of 36 patients with double-level spondylolisthesis treated with TLIF were included and divided into L3/L4 double spondylolisthesis group and L4/L5 double spondylolisthesis group according to the site of spondylolisthesis. The sagittal parameters of the patients were measured by standing anteroposterior and lateral X-rays of the whole spine, and the visual analogue scale (VAS) for lumbar and lower limb pain, Japanese Orthopaedic Association (JOA), and Oswestry Disability Index (ODI) were recorded. The imaging parameters and clinical parameters of the patients before surgery, after surgery, and at the last follow-up were compared and statistically analyzed.

**Results:**

A total of 36 patients were included in the study and all had sagittal imbalance. Among them, there were 21 cases of L3 and L4 spondylolisthesis, 6 males and 15 females, with an average age of 64.7 ± 9.4 years; there were 15 cases of L4 and L5 spondylolisthesis, 4 males and 11 females, with an average age of 66.5 ± 8.0 years. 36 patients completed the operation, the operation time was 190.28 ± 6.12 min, and intraoperative blood loss was 345 ± 11 ml. Compared with preoperative, there were significant differences in SVA, TPA, T1-SPi, LL, PT, SS, PI-LL, SD, SA, and SP between patients after surgery and at the last follow-up (*P* < 0.05). Compared with preoperative, VAS score, JOA score, and ODI index of waist and lower limbs were significantly improved after the operation and at the last follow-up, and there was a significant difference (*P* < 0.05).

**Conclusion:**

TLIF can effectively relieve the symptoms of patients with continuous double-level lumbar spondylolisthesis, restore lumbar lordosis and sagittal spinal sequence, and improve the quality of life of patients.

## Introduction

Lumbar spondylolisthesis (LS) refers to the relative sagittal slip of the adjacent upper lumbar spine relative to the lower lumbar spine [1, 2]. Compared with single-level spondylolisthesis, the incidence of double-level spondylolisthesis is rare, with the incidence of double-level degenerative spondylolisthesis ranging from 5 to 12% [3]and the incidence of double-level isthmic spondylolisthesis ranging from 0.3% to 1.48% [4], while continuous double-level spondylolisthesis predominates in double-level spondylolisthesis [5]. In recent years, most scholars have reported the imaging features of double-level lumbar spondylolisthesis, and the results showed that compared with single-level lumbar spondylolisthesis, patients with isthmic or degenerative double-level lumbar spondylolisthesis are usually accompanied by greater PI, C7 inclination angle and loss of lower lumbar lordosis, and patients will improve the forward inclination of the trunk through pelvic retroversion and compensatory flexion of the hip and knee joints [3, 4, 6]. This sagittal imbalance of anterior trunk inclination has been shown by most scholars to be closely related to lower back pain and seriously affects the quality of life of patients [7, 8].

Lumbar spondylolisthesis is often treated with surgical intervention when conservative treatment is ineffective. Although the treatment modalities and surgical strategies for spondylolisthesis remain controversial, most physicians tend to perform interbody fusion at the same time as laminar decompression given that double-level spondylolisthesis has a more severe spinal stenosis and poor interbody stability caused by extensive decompression of the lamina compared with single-level spondylolisthesis [2, 9, 10]. Therefore, the surgical goals of double-level spondylolisthesis not only include nerve root decompression to improve the patient's clinical symptoms but also require correction of this significant sagittal imbalance by reduction and fusion, thus ensuring the long-term outcome of the surgery [1]. In this study, we reviewed consecutive double-level lumbar spondylolisthesis patients admitted to our hospital to analyze the clinical efficacy of TLIF in the treatment of double-level lumbar spondylolisthesis sagittal imbalance, and to provide a reference for clinical decision-making.

## Methods

The clinical data of consecutive double-level lumbar spondylolisthesis patients treated with TLIF in our hospital between January 2015 and May 2021 were retrospectively analyzed. The study was approved by the Ethics Committee of our hospital, and written informed consent from participants was received. The inclusion criteria were as follows: (1) consecutive double-level lumbar spondylolisthesis was diagnosed on imaging, and there were corresponding imaging clinical manifestations; (2) Surgical treatment was performed in our hospital, and the surgical approach was two-level TLIF; (3) complete preoperative, postoperative, and final follow-up of the relevant clinical and imaging data, and follow-up was more than 1 year. Exclusion criteria included: (1) other types of double-level spondylolisthesis, including continuous double-level spondylolisthesis and mixed anteroposterior double-level spondylolisthesis; (2) patients who had previously undergone lumbar surgery or a second revision surgery; (3) double-level spondylolisthesis combined with other causes of sagittal and/or coronal imbalance of the spine, including trauma, infection, tumor, lateral spondylolisthesis, kyphotic deformity due to congenital developmental deformity, and/or scoliosis > 10°.

Preoperative anteroposterior and lateral X-ray examinations of the whole spine were performed in all patients to observe the lumbar spondylolisthesis segments, which were divided into L3/L4 spondylolisthesis group and L4/L5 spondylolisthesis group according to the spondylolisthesis site.

### Surgical procedure

After strict conservative treatment failed to relieve the patient's clinical symptoms, double-level transforaminal lumbar interbody fusion (TLIF) was performed in our spinal surgery department. Following general anesthesia, the patient was placed in a prone position. A conventional lumbar midline incision was used, and after the incision of the skin and subcutaneous tissue, the paravertebral muscles were dissected subperiosteally using an electrocautery knife and pedicle screws were placed. Resection of the facet joints and standard laminar decompression were performed on the side of the patient's main symptoms and/or significant facet joint hyperplasia, nerve roots were released, the resected facet joints and laminae were removed from the soft tissues, and bone particles were bitten with rongeurs for future use. Following the completion of discectomy and endplate manipulation, the reduction was lifted with bilateral rods. Autologous bone particles and CAGE with autologous bone particles were placed in the intervertebral space, and compression pedicle screws were used to reconstruct lumbar lordosis. The size of the CAGE is determined empirically. After the operation, the drainage tube was routinely placed on the decompression side, and if bilateral decompression was performed, bilateral drainage tubes were placed.

### Data collections

Perioperative conditions of all patients, including gender, age, and body mass index (BMI), were retrospectively analyzed. Functional improvement was assessed by visual analogue scale (VAS) for lumbar and lower extremity pain, Japanese Orthopaedic Association (JOA), and Oswestry disability index (ODI) before surgery, after surgery, and at the last follow-up.

All cases underwent standing anteroposterior and lateral X-rays of the whole spine before surgery, after surgery, and at the last follow-up, and all radiographic parameters were measured by two experienced spine surgeons using Surgimap and averaged to give results. Radiographic parameters included, Overall parameters: sagittal vertical axis (SVA), T1 pelvic angle (TPA), T1 spinopelvic inclination (T1-SPi), thoracic lumbar angle (TLA); local parameters: thoracic kyphosis (TK), lumbar lordosis (LL); spinopelvic parameters: pelvic incidence (PI), pelvic inclination (PT), sacral inclination (SS), pelvic incidence minus lumbar lordosis (PI-LL); spondylolisthesis parameters: slip distance (SD), slip angle (SA), slip percentage (SP). The presence of sagittal balance was determined based on the horizontal distance from the C7 plumb line to the posterosuperior border of S1 (SVA), and the sagittal imbalance was considered if the horizontal distance exceeded 40 mm, the kyphotic angle was negative and the lordotic angle was positive.

### Statistical analysis

The SPSS 21.0 software (Chicago, IL, USA) was applied for statistical analysis. Continuous variables were expressed as mean ± standard deviation and analyzed by the Kirmogrov-Smirnov test for normality assessment. Differences were analyzed by unpaired t-test or Mann–Whitney U test. Categorical variables were analyzed by chi-square test. *P* < 0.05 was considered a statistical significance.

## Results

**Fig. 1:**
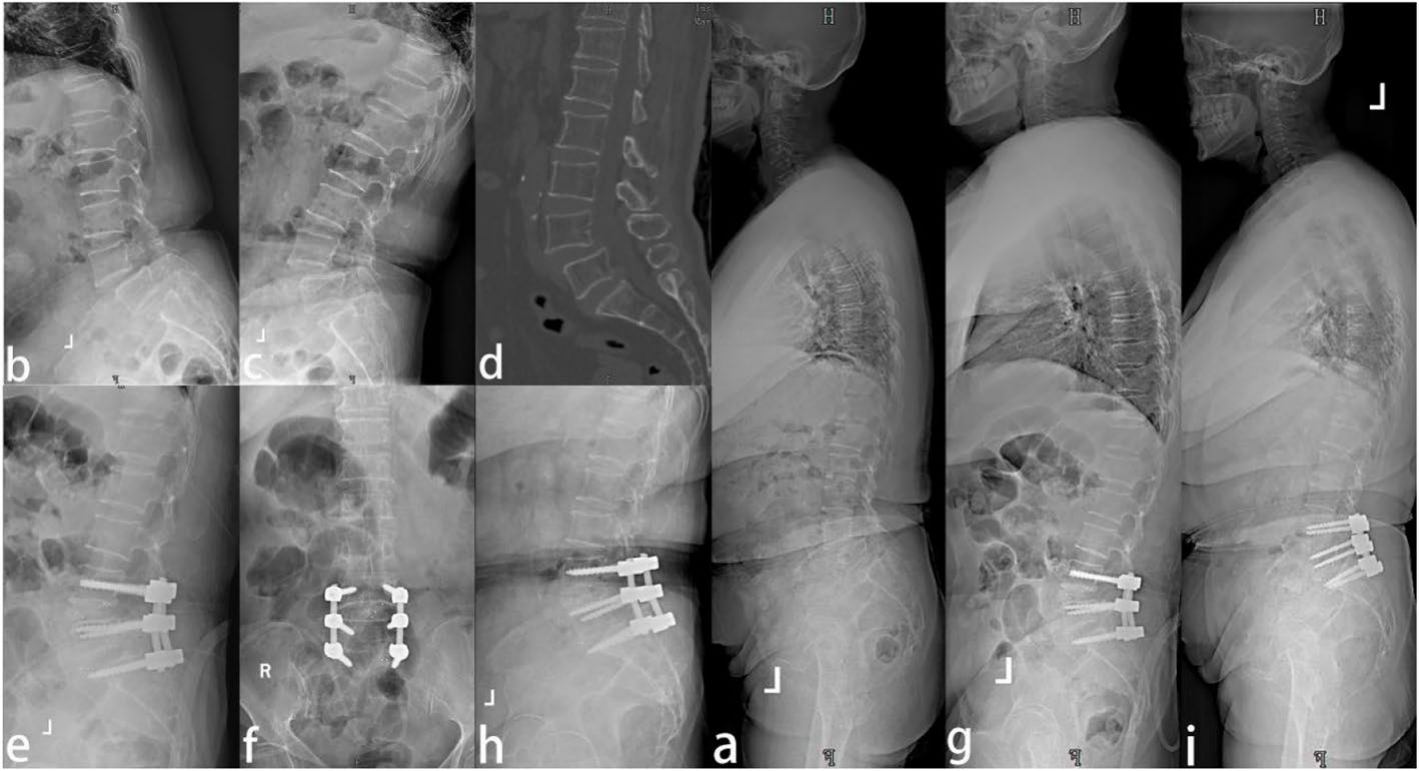
A 53-year-old female patient with L4/L5 two-level lumbar spondylolisthesis **a**, **b**, **c** refers to the preoperative lumbar lateral and whole spine lateral X-ray; **d** refers to the lumbar CT sagittal reconstruction, showing the anterior slippage of the vertebral body; **e**, **f**, **g** refers to the lumbar lateral and whole spine lateral X-ray after two-level unilateral TLIF, which shows that the sagittal imbalance is significantly improved; **h**, **i** refers to the satisfactory internal fixation position during the follow-up at 12 months after operation, and no loss of sagittal correction is observed

A total of 36 patients were included in this study, including 21 patients with L3/L4 double-level spondylolisthesis (18 with degenerative spondylolisthesis and 5 with isthmic spondylolisthesis), 6 males and 15 females, with a mean age of 64.7 ± 9.4 years and mean BMI of 26.5 ± 4.3 kg/m^2^, 16 patients complained of low back and leg pain, and 5 patients complained of low back pain with intermittent claudication; 15 patients with L4/L5 double-level spondylolisthesis (5 with degenerative spondylolisthesis and 10 with isthmic spondylolisthesis), 4 males and 11 females, with a mean age of 66.5 ± 8.0 years and mean BMI of 24.9 ± 3.5 kg/m^2^, of whom 8 patients complained of low back and leg pain and 7 patients complained of low back pain with intermittent claudication. 36 patients completed the operation, the average operation time was 190.28 ± 6.12 min, and the average intraoperative blood loss was 345 ± 11 ml. Two patients who presented with numbness of lower limbs on a postoperative day received nutritional nerve drugs and were observed dynamically. The numbness of the lower limbs was improved obviously after 7 days. Typical cases are shown in Fig. 1.

Details of radiological parameters preoperative, postoperative, and at the final follow-up were available for 36 patients (Table 1, Table 2). And all patients had a sagittal imbalance of the spine preoperatively, and the mean SVA was 77.58 ± 24.81 mm. Compared with preoperative, SVA, TPA, T1-SPi, LL, PT, SS, PI-LL, SD, SA, and SP were significantly improved after the operation and at the last follow-up *(P* < 0.05), while TLA, PI, and TK were not significantly changed (*P* > 0.05). Compared with postoperative, there was no significant difference in imaging parameters at the last follow-up (*P* > 0.05). In L3/L4 double-level spondylolisthesis, preoperative SD (5.83 ± 1.57 mm, 6.35 ± 2.46 mm), SA (4.55 ± 2.85°, 1.76 ± 5.34°), improved to postoperative SD (0.21 ± 1.14 mm, 0.75 ± 1.32 mm), SA (7.41 ± 2.75°, 6.65 ± 3.93°); in L4/L5 double-level spondylolisthesis, preoperative SD (8.43 ± 5.72 mm, 11.26 ± 3.91 mm), SA (3.97 ± 5.40°, 4.93 ± 5.23°), improved to postoperative SD (0.45 ± 1.57 mm, 2.37 ± 2.24 mm), SA (6.87 ± 5.32°, 9.73 ± 5.61°). In L3/L4 double-level spondylolisthesis and L4/L5 double-level spondylolisthesis, preoperative SVA (72.12 ± 22.91 mm, 85.21 ± 25.84 mm), TPA (25.65 ± 5.47°, 28.75 ± 7.21°), T1-SPi (-2.93 ± 3.84°, -0.53 ± 3.24°) were improved to postoperative SVA (24.50 ± 17.15 mm, 33.78 ± 16.18 mm), TPA (16.37 ± 3.88°, 17.92 ± 4.53°), T1-SPi (-4.79 ± 2.74°, -4.77 ± 6.07°). In L3/L4 double-level spondylolisthesis and L4/L5 double-level spondylolisthesis, it was improved from preoperative LL (40.11 ± 10.43°, 50.97 ± 13.18°), PI-LL (17.11 ± 7.04, 20.35 ± 8.26), PT (27.35 ± 4.11, 28.81 ± 4.77) to postoperative LL (43.75 ± 12.58°, 53.58 ± 13.17°), PI-LL (14.34 ± 5.17, 16.90 ± 7.83), PT (21.36 ± 4.56°, 22.79 ± 6.22°). Compared with postoperative, the above imaging parameters were well maintained at the final follow-up.

**Table 1:** Preoperative, postoperative and follow-up slippage parameters

	Preoperative	Postoperative	Final follow-up	*P* value	Preoperative	Postoperative	Final follow-up	*P* value
L3 and L4 double-level spondylolisthesis								
	L3 spondylolisthesis				L4 spondylolisthesis			
SD(mm)	5.83 ± 1.57	0.21 ± 1.14	0.25 ± 1.25	< 0.001	6.35 ± 2.46	0.75 ± 1.32	0.72 ± 1.48	< 0.001
SA(°)	4.55 ± 2.85	7.41 ± 2.75	7.62 ± 2.80	< 0.001	1.76 ± 5.34	6.65 ± 3.93	6.78 ± 4.17	< 0.001
SP(%)	14.73 ± 4.47	2.39 ± 1.74	2.29 ± 2.19	< 0.001	15.44 ± 6.01	3.64 ± 2.13	3.35 ± 2.03	< 0.001
L4 and L5 double-level spondylolisthesis								
	L4 spondylolisthesis				L5 spondylolisthesis			
SD(mm)	8.43 ± 5.72	0.45 ± 1.57	0.65 ± 1.32	< 0.001	11.26 ± 3.91	2.37 ± 2.24	2.53 ± 1.54	< 0.001
SA(°)	3.97 ± 5.40	6.87 ± 5.32	7.05 ± 4.70	0.038	4.93 ± 5.23	9.73 ± 5.61	10.03 ± 5.17	0.019
SP(%)	19.47 ± 7.35	3.57 ± 1.07	3.53 ± 1.33	< 0.001	25.37 ± 8.92	6.35 ± 5.83	6.17 ± 5.03	< 0.001

*P* Preoperative vs. Final follow-up

**Table 2:** Preoperative, postoperative and final follow-up sagittal parameters

	Preoperative	Postoperative	Final follow-up	*P* value	Preoperative	Postoperative	Final follow-up	*P* value
	L3 and L4 double-level spondylolisthesis				L4 and L5 double-level spondylolisthesis			
PI(°)	57.87 ± 8.71	57.70 ± 8.33	57.81 ± 8.71	0.705	71.31 ± 12.33	71.38 ± 15.96	71.07 ± 14.80	0.818
PT(°)	27.35 ± 4.11	21.36 ± 4.56	21.32 ± 4.29	< 0.001	28.81 ± 4.77	22.79 ± 6.22	22.80 ± 8.10	0.001
SS(°)	30.89 ± 9.10	36.74 ± 10.13	36.10 ± 9.68	< 0.001	41.13 ± 12.08	48.19 ± 15.36	48.07 ± 15.60	0.001
PI-LL	17.11 ± 7.04	14.34 ± 5.17	14.09 ± 4.82	0.032	20.35 ± 8.26	16.90 ± 7.83	16.77 ± 7.96	0.022
LL(°)	40.11 ± 10.43	43.75 ± 12.58	43.43 ± 12.75	0.034	50.97 ± 13.18	53.58 ± 13.17	53.39 ± 13.14	0.024
TK(°)	-16.76 ± 7.93	-18.93 ± 6.79	-18.88 ± 6.45	0.090	-26.25 ± 9.86	-26.47 ± 9.14	-26.11 ± 9.01	0.451
TPA(°)	25.65 ± 5.47	16.37 ± 3.88	16.37 ± 3.71	< 0.001	28.75 ± 7.21	17.92 ± 4.53	17.04 ± 4.53	< 0.001
TLA(°)	13.77 ± 8.75	10.38 ± 6.06	10.32 ± 6.44	0.067	17.51 ± 8.80	13.06 ± 8.11	12.99 ± 7.93	0.139
T1-SPi(°)	-2.93 ± 3.84	-4.79 ± 2.74	-4.74 ± 2.73	0.009	-0.53 ± 3.24	-4.77 ± 6.07	-4.86 ± 6.00	0.031
SVA(mm)	72.12 ± 22.91	24.50 ± 17.15	24.68 ± 18.63	< 0.001	85.21 ± 25.84	33.78 ± 16.18	32.19 ± 14.94	< 0.001

*P* Preoperative vs. Final follow-up

The VAS score, JOA score, and ODI index of the waist and lower limb preoperative, postoperative, and during the final follow-up are shown in Table 3 Compared with preoperative, the VAS score, JOA score, and ODI index of waist and lower limbs of patients were significantly improved postoperative and at the final follow-up, and there was a significant difference (*P* < 0.05); compared with postoperative, the VAS score, JOA score and ODI index of patients were further improved at the final follow-up, and the difference had statistical significance (*P* < 0.01).

**Table 3:** Preoperative, postoperative and final follow-up function outcomes

	Preoperative	Postoperative	Final follow-up	*P* value
VAS of low back pain	5.1 ± 1.7	4.3 ± 1.5	2.7 ± 1.3	< 0.001
VAS of lower limbs pain	5.2 ± 1.7	2.7 ± 0.9	1.6 ± 1.1	< 0.001
JOA	14.9 ± 4.4	17.1 ± 3.5	25.6 ± 2.3	< 0.001
ODI	34.0 ± 7.5	21.7 ± 5.1	18.9 ± 4.8	< 0.001

*P* Preoperative vs. Final follow-up

## Discussion

Wiltse et al. [11] classified lumbar spondylolisthesis into dysplasia, spondylolysis, degeneration, trauma, and pathology based on the cause of the disease. In previous studies, it has been observed that degenerative and isthmic spondylolisthesis are more common in double-level spondylolisthesis; degenerative double-level spondylolisthesis is more common compared with isthmic spondylolisthesis [3, 4]. Although there is no consensus on the pathogenesis of lumbar spondylolisthesis, some scholars believe that multilevel degenerative lumbar spondylolisthesis may be associated with factors such as advanced age, high BMI, changes in the direction of the lumbar facet joints, degenerative changes in the intervertebral discs, ligament and paravertebral muscle dysfunction and high PI, and high PI, LL, and SS will bring greater shear forces to the lumbosacral junction and make isthmus stress greater, so high PI is considered to be a predictor of double-level lumbar spondylolisthesis [12–14]. In double-level degenerative spondylolisthesis, there were more females than males, and it was mainly considered that it may be related to pregnancy, systemic joint laxity, and hormones; and the affected segment was mainly L3/L4 spondylolisthesis, which was mainly considered to be related to the denser lumbosacral ligaments [15]. In double-level isthmic spondylolisthesis, there were more males than females, and most of them had related factors such as strenuous exercise and heavy physical work, and L4/L5 spondylolisthesis was predominant in the affected segments, which were mainly considered to be associated with chronic fatigue fractures in the lumbosacral region as a stress concentration in the lumbar spine [1, 9]. In this study, there were 21 cases of double-level spondylolisthesis at L3/L4, including 18 cases of degenerative spondylolisthesis and 15 cases of double-level spondylolisthesis at L4/L5, including 10 cases of isthmic spondylolisthesis, which were consistent with the results of previous studies.

When viewed from the lateral side, there are five physiological curves in the human spine, connected superiorly to the skull base and inferiorly to the pelvis, which together constitute the sagittal sequence of the spine. Normal sagittal sequences allow the human body to maintain an upright state through minimal energy expenditure and load, but this sagittal balance will be broken as the spine changes retrograde. Ferrero et al. [3] compared the characteristics of sagittal parameters in single-level versus double-level degenerative spondylolisthesis and concluded that PI, PT, and C7 inclination angles were significantly higher in double-level spondylolisthesis compared with single-level spondylolisthesis, and lower lumbar lordosis loss was more significant in the double-level spondylolisthesis group. Du et al. [4] proposed that although there are differences in the pathogenesis between double-level isthmic spondylolisthesis and double-level degenerative spondylolisthesis, from the nature of double-level spondylolisthesis, patients with continuous double-level isthmic spondylolisthesis have lower disc height and more significant forward slippage, which may also be the main reason for their sagittal imbalance. Therefore, compared with single-level lumbar spondylolisthesis, either degenerative or isthmic double-level lumbar spondylolisthesis presents with significant sagittal imbalance, including loss of lower lumbar lordosis, anteversion of the trunk, and compensatory flexion of the hip and knee joints, and this sagittal imbalance seriously affects the quality of life of patients [7, 8]. Schwab et al. [16] showed that in adult patients with spinal deformity, ODI scores were higher when PT > 22°, SVA > 47 mm, and PI-LL > 11°, and mismatch between PI and LL was significantly associated with patient quality of life. Lafage et al. [17] also proposed that an increase in PT is closely related to a worsening of the patient's quality of life and that a combination of SVA and PT is needed to assess sagittal imbalance. Because there is no comprehensive assessment method for sagittal imbalance, Schwab classification is the main method for assessing sagittal imbalance, and TPA, T1-SPi, and TLA global sagittal parameters are added to assess the changes of imaging parameters before, after, and at the last follow-up in double-level lumbar spondylolisthesis. In a geometric relationship, TPA = PT + T1-SPi, reflects both pelvic rotation and spinal tilt, although there is no agreement on the orthopedic target value of TPA in adult patients with spinal deformity, TPA values have been identified to be closely related to the patient's quality of life, and the advantage of TPA is that it is not changed by the patient's postural changes, and its TPA values remain unchanged regardless of whether there are compensatory changes in the patient's pelvis or hip and knee joints [18, 19]; TLA mainly responds to segmental compensatory changes in degenerative diseases from proximal to distal segments, thereby assessing thoracolumbar segmental changes [20]. The results of this study showed that compared with those before surgery, TPA and T1-SPi in L3/L4 and L4/L5 spondylolisthesis groups were significantly improved after surgery and at the last follow-up compared with those before surgery, and there was statistical significance; while TLA was improved from 13.77 ± 8.75° preoperatively to 10.38 ± 6.06° postoperatively in L3/L4 spondylolisthesis group, and from 17.51 ± 8.80° preoperatively to 13.06 ± 8.11° postoperatively in L4/L5 spondylolisthesis group, and there was no statistical difference. The authors concluded that postoperative SVA, TPA, T1-SPi, and PT recovered well, trunk anteversion and pelvic retroversion recovered, but there was no significant change between thoracolumbar segments, and thus concluded that preoperative two-level spondylolisthesis showed a state of instability locally in the lumbar spine, compensatory changes occurred through trunk anteversion superiorly and pelvic retroversion inferiorly, while thoracolumbar segmental compensation was less, so although TLA improved postoperatively, it was not statistically significant, and the above parameter changes may also be related to the compensatory mechanism of the elderly during degeneration. For an in-depth understanding of imaging parameters, we can provide corresponding guidance during orthopedic procedures [21].

Interbody fusion is widely accepted due to the greater severity of spinal stenosis and poor intervertebral stability in double-level lumbar spondylolisthesis [22, 23]. Common interbody fusion methods include the anterior/trapezius approach (ALIF/OLIF) and posterior approach (PLIF/TLIF). The advantages of ALIF/OLIF are that it can maintain the tension of posterior spinal structures by preserving the posterior ligamentous complex, and it is easier to completely remove the intervertebral disc and thus place a larger cage; however, most complications of ALIF/OLIF are also related to its approaches, such as potential visceral injury, retrograde ejaculation and sympathetic dysfunction [24]. Compared with ALIF/OLIF, PLIF/TLIF paravertebral muscle dissection can anatomically bring greater iatrogenic trauma, but it can provide a wider and direct vision of the surgical field and provide conditions for thorough nerve root decompression. Compared with PLIF, the transforaminal approach makes intraoperative traction between the dural sac and nerve roots less necessary, which can avoid the possibility of dural sac and nerve root injury to some extent [25]. TLIF requires resection of at least one facet joint, and can directly decompress the lateral recess stenosis, achieve interbody dynamic distraction reduction through pedicle screws and cages, and better restore lumbar lordosis and sagittal spinal sequences [4]. Considering the sagittal features of double-level lumbar spondylolisthesis, the surgical emphasis includes not only the management of spondylolisthesis but also the correction of the overall sagittal spinal sequence. It is prudent to consider how distraction reduction of localized spondylolisthesis impacts the overall sagittal spinal sequence and to define an appropriate surgical target for the patient before surgery.

Given that patients with double-level spondylolisthesis are generally older, have more comorbidities, and have limited tolerance to the degree of surgery, our surgical strategy was not developed to target ideal sagittal parameters for peers. For spondylolisthesis, we recommend that reduction be accomplished as far as possible rather than pursuing a perfect anatomical reduction because forced reduction may result in relative displacement of the nerve roots causing injury. The results of this study showed that the spondylolisthesis parameters (SD, SA, SP) were significantly improved after TLIF in both L3/L4 spondylolisthesis and L4/L5 spondylolisthesis, and the spondylolisthesis parameters were well maintained at the last follow-up, indicating that double-level TLIF was effective in the appropriate reduction and intervertebral distraction of double-level spondylolisthesis. Schwab et al. [26] suggested that adult patients with spinal deformity should be targeted at LL = PI ± 9°, PT < 20°, and SVA < 50 mm, while Soroceanu et al. [27] also proposed that complications such as adjacent segment degeneration and internal fixation device loosening may result if the postoperative sagittal parameters fail to meet the revised classification of SRS-Schwab classification. The results of this study showed that SVA, TPA, and T1-SPI were significantly improved after TLIF in the L3/L4 and L4/L5 spondylolisthesis groups, indicating that TLIF significantly improved the state of trunk inclination based on correcting the spondylolisthesis distance and angle; while PI-LL improved from 17.11 ± 7.04 preoperatively to 14.34 ± 5.17 postoperatively in the L3/L4 spondylolisthesis group; and from 20.35 ± 8.26 preoperatively to 16.90 ± 7.83 postoperatively in the L4/L5 group. Among them, PI-LL did not meet the SRS-Schwab modified classification, but the overall sagittal parameters achieved satisfactory results. The authors analyzed that the degree of PI and LL matching is specific and cannot be measured by the same criterion, which is basically consistent with the findings of Diebo et al. [28]. After fixed fusion and appropriate restoration of LL, the sagittal parameters (SVA, TPA, T1-SPi) were also significantly improved, and the author concluded that the sagittal parameters were dynamic changes, and the instability of the lower lumbar spine or lumbosacral intervertebral space was improved by surgery, and the lordosis and sagittal sequences were also restored, so we believed that appropriate restoration of lumbar lordosis according to the Schwab modification classification could obtain satisfactory sagittal changes. At the same time, compared with preoperative, the VAS score, JOA score, and ODI index of the lumbar region and lower extremities of the patients were significantly improved after surgery and at the last follow-up, indicating that two-level TLIF is effective in reconstructing lumbar lordosis, restoring sagittal sequence, improving patient function, and improving patient quality of life.

Our experience suggests that most patients with double-level spondylolisthesis have previously been diagnosed because various factors have not been paid attention to, and lumbar and lower limb symptoms further worsen with the progression of the disease, showing more significant facet joint hyperplasia, spinal canal or lateral recess stenosis and significant sagittal imbalance on imaging, while TLIF can directly remove the facet joints and decompress the lateral recess while restoring the lumbar lordosis angle. The authors believe that the change of spondylolisthesis parameters is the basis of the change of sagittal parameters, and by correcting the local vertebral slippage, the recovery of intervertebral space height and angle, that is, improving the sagittal compensation of the spine, the sagittal sequence balance of the spine can be better restored. We recommend that reconstruction of lumbar lordosis be accomplished by placing CAGE of appropriate vertebral body size to distract the anterior vertebral space and placing pedicle screws. Global sagittal sequence balance was restored by changes in local sagittal parameters.

However, this study is a single-center institutional review, the sample size is small, there is still a lack of control of different surgical procedures, there are still defects, and the shortcomings can be further improved in the future, to clarify the clinical efficacy of TLIF in the treatment of double-level spondylolisthesis.

## Conclusion

Whether it is L3/L4 double-level spondylolisthesis or L4/L5 double-level spondylolisthesis, double-level TLIF can effectively relieve the symptoms of patients and improve the functional status of patients, while improving the sagittal sequence of the spine and improving the quality of life of patients based on restoring spondylolisthesis, and it is safe and effective.

## Data Availability

The data sets generated and analyzed during the current study are not publicly available due to restrictions on ethical approvals involving patient data and anonymity but can be obtained from the corresponding author on reasonable request.

## Abbreviations

LL: Lumbar lordosis
ODI: Oswestry disability index
PI: Pelvic incidence
PT: Pelvic tilt
PI-LL: Pelvic incidence minus lumbar lordosis
SS: Sacral slope
SD: Slip distance
SA: Slip angle
SVA: Sagittal vertical axis
TLIF: Transforaminal lumbar interbody fusion
TK: Thoracic kyphosis
TPA: T1 pelvic angle
T1-SPi: T1 spinopelvic inclination
TLA: Thoracic lumbar angle
